# Dietetics Program Directors in the United States Support Teaching Vegetarian and Vegan Nutrition and Half Connect Vegetarian and Vegan Diets to Environmental Impact

**DOI:** 10.3389/fnut.2019.00123

**Published:** 2019-08-14

**Authors:** Irana W. Hawkins, A. Reed Mangels, Robert Goldman, Richard J. Wood

**Affiliations:** ^1^Department of Nutrition, University of Massachusetts Amherst, Amherst, MA, United States; ^2^Doctoral Programs in Public Health, School of Health Sciences, Walden University, Minneapolis, MN, United States; ^3^Math and Computational Sciences, Simmons University, Boston, MA, United States

**Keywords:** vegetarian nutrition, vegan nutrition, dietetics education, dietetics practice, plant-based nutrition

## Abstract

Registered Dietitian Nutritionists (RDNs) are clinicians trained in the application of food, nutrition, and dietetics. Vegetarians and vegans have a lower risk of many nutrition-related chronic diseases that are epidemic while vegetarian and vegan diets are associated with reduced environmental impact. Despite this strong diet-disease and diet-environment connection, it is not known if dietetics students are taught the principles of vegetarian and vegan nutrition. The overarching goal of our study was to investigate curricular practices in accredited dietetics training programs in the United States (U.S.) including (1) the prevalence and perceived importance of vegetarian and vegan nutrition instruction and (2) if program directors connect vegetarian and vegan diets to climate change mitigation and resource conservation. Primary data were collected by way of a cross-sectional, Internet-based survey. All Accreditation Council for Education in Nutrition and Dietetics (ACEND) program directors in the U.S. (*N* = 574) were sent a 37-question survey and invited to participate in the study. Outcome measures included the prevalence of vegetarian and vegan nutrition instruction, quantifying if relationships exist among variables, and the frequency of connecting vegetarian and vegan diets to environmental impact. Descriptive and inferential statistics were utilized. Respondents (*n* = 205) indicated that over 51% of programs teach vegetarian nutrition while 49% teach vegan nutrition. There were significant differences between program type and the prevalence of vegetarian (*p* = 0.00005) and vegan (*p* = 0.00005) nutrition instruction. Over 90% of program directors believe that vegetarian and vegan nutrition should be taught. Over 50% of programs identify the connection between vegetarian and vegan diets in climate change mitigation and resource conservation. Most ACEND program directors believe vegetarian and vegan nutrition should be taught and half connect diet to environmental concern. Nevertheless, there is a discrepancy between beliefs and practice behaviors. These results suggest the need for increased collaboration and the use of novel techniques that better incorporate vegan and vegetarian nutrition throughout dietetics education.

## Introduction

Many of the leading causes of mortality in the U.S. are diet-related, with heart disease the number one killer and cancer, stroke, and diabetes among the top seven causes of mortality (1, 2). More than two-thirds of adults in the U.S. are overweight or obese (3). Almost 20% of children and adolescents in the U.S. are obese; almost 6% are classified as severely obese (4). Overweight and obesity are risk factors for heart disease, diabetes, high blood pressure, stroke, osteoarthritis, and certain cancers. Type 2 diabetes (T2D) is an epidemic with 1.5 million new cases per year in adults in the U.S. (5). In addition to overweight/obesity, risk factors for cancer include lack of physical activity and unhealthy dietary patterns including a high consumption of red or processed meats and a very low intake of whole grains, fiber, fruit and vegetables (6).

A growing body of literature demonstrates that vegetarians and vegans have a lower risk of many diet-related chronic diseases and that vegetarian and vegan diets are effective in the treatment of these diseases (7). Meta-analyses have shown that vegetarian diets can significantly lower blood pressure (8) and BMI (body mass index) (9). Additionally, vegetarians have a 29% lower mortality from ischemic heart disease (10) and an 8% lower risk of total cancer (9) compared to omnivores. In studies comparing different types of vegetarians and non-vegetarians, vegans have the lowest BMI (11), blood pressure (12), and prevalence of diabetes (11). Vegetarian diets have been successfully used to treat T2D (13, 14) and have significant benefits related to weight reduction compared to non-vegetarian diets (15). Additionally, components of vegetarian and vegan diets including phytonutrients (16), dietary nitrates (17), and dietary fiber (18) have demonstrated impressive beneficial health outcomes. Although the Academy of Nutrition and Dietetics encourages fiber intake from a wide variety of whole plant foods, only 5% of Americans achieve an adequate intake (18).

The 2015–2020 *Dietary Guidelines for Americans* recommends “a healthy vegetarian eating pattern” as one of three “healthy eating patterns that can be adapted based on cultural and personal preferences” (19). The position statement on vegetarian diets of the Academy of Nutrition and Dietetics (the world's largest organization of food and nutrition professionals) endorses appropriately planned vegetarian and vegan diets which are considered safe and appropriate for all stages of the lifecycle and recognizes their therapeutic role in chronic disease prevention and management (7). It is also important to underscore the data implicating meat in adverse health outcomes. The International Agency for Research on Cancer classified processed meat as carcinogenic to humans while red meat was classified as possibly carcinogenic (20). Epidemiological studies implicate red meat in diabetes risk as well as cardiovascular mortality (21, 22).

The Academy of Nutrition and Dietetics credentials Registered Dietitian Nutritionists (RDNs) and Nutrition and Dietetic Technicians, Registered (NDTRs) in the U.S through the Commission on Dietetic Registration (23). RDNs and NDTRs are the healthcare providers solely trained in the application of food, nutrition, and dietetics so to protect public health and promote well-being (24). The work of RDNs and NDTRs is interdisciplinary, spanning a multitude of career paths and disciplines that includes but is not limited to healthcare practice and healthcare administration; growing food, preserving food, and all aspects of agriculture that includes local, regional, and global food systems; technology and social media; research; urban planning; food security and global sustainable development; epidemiology and public health; planetary health and biodiversity; and education spanning from early care to higher education.

Despite the impressive evidenced-based data supporting vegetarian and vegan diets in chronic disease management and prevention, several studies suggest that dietetics practitioners may have knowledge deficits and lack confidence in the area of vegetarian and vegan nutrition. For example, 23% of Missouri dietitians demonstrated knowledge deficits with regard to the statement “the only high quality proteins are animal proteins” (25). Half of dietitians surveyed in 2012 stated that animal products were *essential* for a healthy diet (25). Although more than 70% of Canadian healthcare providers including dietitians were aware that plant-based diets could be used to manage T2D, less than one-third recommended these diets (26). Hence, Bandura's constructs of self-efficacy may be particularly useful to dietitians in the realm of applying vegetarian and vegan nutrition in practice (27, 28). As Bandura explains, the processes of developing hands-on skills and mastery experiences, modeling the behaviors, increasing performance standards and minimizing anxiety when facing challenges or setbacks can build self-efficacy (27, 28). Self-efficacy was shown to be an important factor in dietitians leading the charge of environmental care (29) and was likely a factor for vegetarian and vegan dietitians that use diet as a climate change mitigation strategy (30).

As the proposed modern-day geological era known as the Anthropocene demonstrates, humans are altering the Earth System in ways that may altogether change the Earth system and its ability to support humanity (31). Steffen and colleagues delineate that if the current human-dominated trajectory of the “Hothouse Earth” does not shift toward a “Stabilized Earth” model, irreversible and dangerous outcomes may ensue—especially for those most vulnerable that have limited resources (31). Anthropogenic greenhouse gas emissions that drive increases in the Earth's surface temperatures are linked to our fossil-fuel based food and animal agriculture systems that have degraded the biosphere along with the constant and massive overuse of natural resources (32).

Furthermore, anthropogenic biodiversity loss and climate change negatively impact the Earth System and the planetary boundaries (33). In fact, Ceballos and colleagues stated that, “The loss of biological diversity is one of the most severe human-caused global environmental problems” (34). The May 6, 2019 press conference of the Intergovernmental Panel on Biodiversity and Ecosystem Services (IPBES) discussed highlights of first intergovernmental report on the global state of biodiversity where over 15,000 scientific publications were scrutinized (35). The IPBES scientists emphasized, “The scale of biodiversity loss is immense, and the sense of urgency indicates we have no time to waste. We need bold action and commitment from local to global levels” (36). Examples of such actions included thoughtful dietary choices (36).

An important body of literature delineates that wholesome plant-based diets including vegetarian and vegan diets can positively impact the natural environment (37–44). Acknowledging that food and agriculture is a major driver of both poor human health and environmental degradation, the recent EAT-Lancet Commission Report calls for a “Great Food Transformation” where food systems produce healthy diets from agricultural processes that nurture the planetary processes which are inextricably tied to human health (45). Their proposed “planetary health diet” emphasizes whole plant foods including fruits, vegetables, whole grains, nuts, and legumes which dominate the recommendations (46).

Dietetics professionals have long advocated for sustainable food systems (47, 48), minimizing food waste (49), supporting biodiversity (48, 50), creating resilient food and water systems (51), and using diet to mitigate climate change (30). Most dietitians surveyed (75%) from a random sample of all credentialed dietitians in the U.S. believe climate change is an important issue (30). Dietitians have also sought to understand the feasibility of incorporating sustainable food systems education into dietetics education (52, 53) while some dietetics education programs emphasize sustainable food systems (54). Lastly, dietitians and other clinicians have been called upon to enact forward-thinking leadership to help individuals and communities protect planetary health with actions such as mindful food choices (55).

Because of (1) the burden of epidemic levels of obesity and T2D and the therapeutic value of vegetarian and vegan diets in chronic disease prevention and management and the associated health care cost savings (40), (2) the urgency of mitigating the impact of food choices in breaching our planetary boundaries in order to maintain planetary health, and (3) the need to correct dietetics-based knowledge and practice deficits for those pursuing careers as RDNs and NDTRs, our research study was conceptualized. It is unclear if program directors of accredited dietetics education programs in the U.S. teach vegetarian and vegan nutrition or if they connect vegetarian and vegan diets to environmental conservation. Thus, the overarching objective of our study was to investigate curricular practices in accredited dietetics training programs in the U.S. including (1) the prevalence and perceived importance of vegetarian and vegan nutrition instruction and (2) if program directors connect vegetarian and vegan diets to climate change mitigation, resource conservation, and reducing impact on the natural environment. This understanding can delineate and bridge gaps in both dietetics education and dietetics practice. Hence, we investigated the curricular practices of Accreditation Council for Education in Nutrition and Dietetics (ACEND) programs in the U.S. ACEND is the accrediting agency of the Academy of Nutrition and Dietetics (23) while the U.S. Department of Education ensures that ACEND meets national standards (56).

In addition to our overarching research goals, we sought answers to numerous research questions including: the presence and type of barriers related to vegetarian and vegan diet instruction (if any); if a relationship exists amongst the characteristics of the university or program (e.g., region of the country, public, private, or religious affiliation, etc.); and the prevalence of vegetarian and vegan nutrition instruction or the associated connections between vegetarian and vegan diets and impact on the natural environment.

Programs accredited by ACEND encompass the educational and supervised practiced-based training experiences that prepare students for careers as dietitians or dietetic technicians (23). Didactic Programs in Dietetics (DPDs) are the undergraduate and graduate dietetics coursework completed before the dietetic internship (DI) which is a supervised practice experience completed after a baccalaureate or graduate degree. Coordinated Programs in Dietetics (CPDs) are undergraduate or graduate level dietetics coursework combined with the supervised practice experience. Dietetic technician programs offer a combined associate's degree and supervised practice experience (23).

## Materials and Methods

### Study Design and Participants

This cross-sectional, Internet-based survey targeted all ACEND-accredited program directors listed on the ACEND website on July 19, 2017. International programs outside of U.S. territories were not included. This study was carried out in accordance with the recommendations of the University of Massachusetts Amherst Institutional Review Board with written informed consent from all subjects. All subjects gave written informed consent in accordance with the Declaration of Helsinki. The protocol was approved by the University of Massachusetts Amherst Institutional Review Board. Program directors represented CPD *n* = 61| DI *n* = 257| DPD *n* = 219| and NDTRs *n* = 37 for a total universe of *N* = 574 programs. Two program directors were responsible for both a DI and a DPD and were instructed to complete one survey for each program if participating.

The target sample size was *n* = 231 from a total universe of 574 ACEND-accredited programs. This target sample size was based on a 5% margin of error for a 95% confidence interval for population percentages. A (worst case) scenario of 50% for population percentages was assumed. In fact, the achieved sample size was *n* = 205, somewhat less than the target of 231. As a consequence, the margin of error for population percentages varied from a low of 4% (when the sample percentage was small or was large) to 5.5% (when the sample percentage was close to 50%).

We were certainly aware of the possibility of non-response bias in our results. We were however, heartened by the fact that the mix of regions represented in our sample is a close match to the mix in the population of programs (Chi-square test of fit: *X*^2^ = 0.73, *p* = 0.867). Similarly, the mix of program types in our sample is a close match to the corresponding mix in the population (Chi-square test of fit: *X*^2^ = 0.3.39, *p* = 0.335). Interestingly, some recent research is debunking the presumed relationship between survey response rates and the extent of non-response bias (57).

### Data Collection

**Figure 1:**
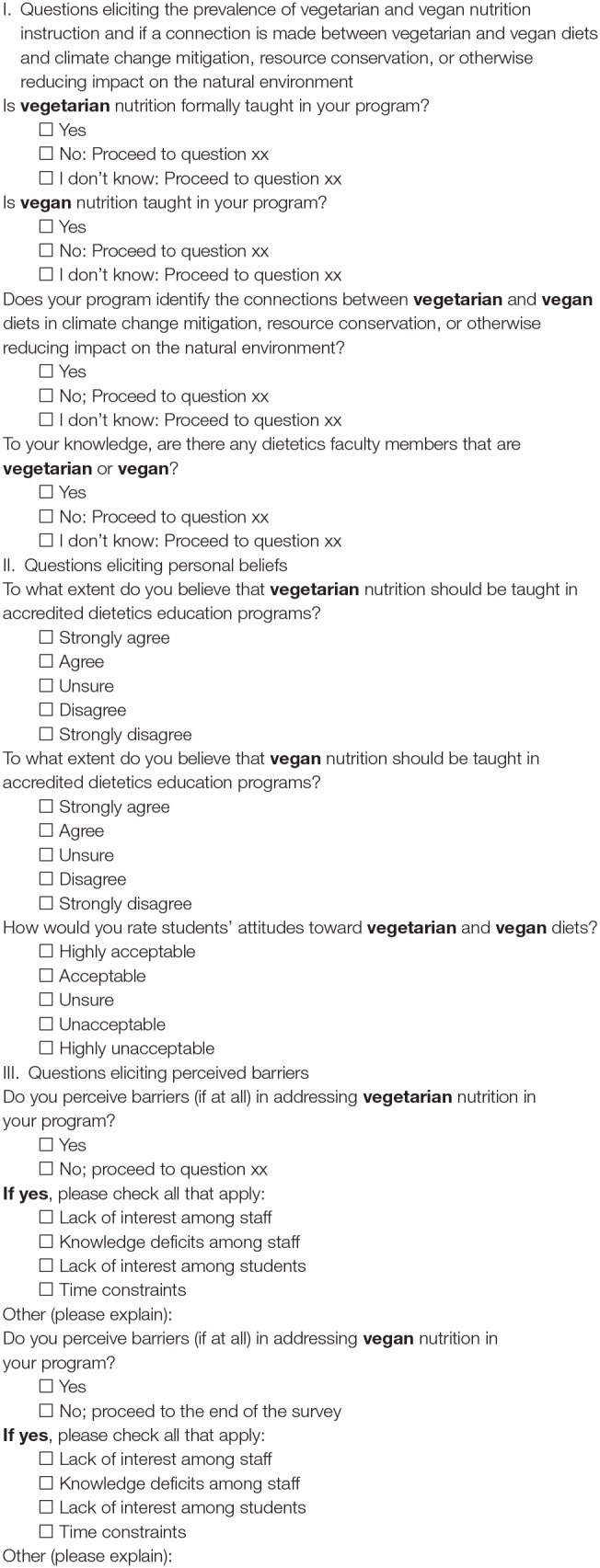
Examples of questions from the survey.

The survey included 37 questions that captured demographic data along with nominal and ordinal questions. An excerpt of our survey is included in Figure 1. As a first-time exploratory study, we could not locate a valid and reliable survey tool that addressed our research goals and questions. Similar to other important exploratory studies that connect seemingly disparate areas of healthcare and public health practice to environmental care (58, 59), we devised our survey tool with carefully planned steps and procedures. First, an informal pilot survey conducted in 2015 critically informed the content of this research study. From there, survey questions were developed in accordance with our research goals and were designed to elicit answers to our research questions. Our survey was then piloted and tested for face validity among seven (*n* = 7) dietetics educators and reviewed extensively by our research team. When revisions were recommended, they were discussed among our research team, tested again, and were then incorporated into the final survey.

The survey was administered online using the SurveyMonkey (San Mateo, CA) research service. Program directors were invited to participate via an email with the subject line entitled, “Survey: Vegetarian and Vegan Nutrition in ACEND Programs” which included the consent form and a link to the survey. Upon providing informed consent, respondents had the option of answering or skipping questions as desired. Automatic skips were also used based on responses to previous questions. It was made clear that all data would remain confidential. Upon completion of the survey, respondents could enter a drawing for one of two $150 checks offered as a gesture of gratitude for their time and efforts. The survey was designed to be completed within 15–30 min.

The survey ran from July 19, 2017 to October 13, 2017 (87 days). Due to the time of year that may be associated with summer and fall holidays coupled with the lower response rates noted amongst other national Internet-based surveys of RDNs in the U.S. (60–63), email reminders about the survey were sent weekly for the first 3 weeks and again at 7 and 8 weeks. Because the response rate was <50% at the end of 8 weeks, a subset of randomly selected non-respondents received a reminder telephone call. We called forty (40) program directors selected using a random number generator from the list of non-respondents. Contacting this subset of forty helped us understand if reminder phone calls were a worthwhile endeavor. Thirty-two (32) potential respondents did not answer and messages were left per our telephone script. None of these program directors responded to the survey. We did not leave a message at two (2) telephone numbers because it was not personal voicemail that corresponded with the respective program director's name. Five (5) program directors answered our call and said they would or would try to complete the survey. Of these, three (3) responded to our survey. There was one (1) wrong number. Because the response rate after the first round of reminder calls was low, this technique was discontinued.

### Data Analysis

The survey data were analyzed using the software package Minitab 18 (College Station, PA). Descriptive statistics involved almost exclusively cross-tabulations. Inference methods were the chi-square test for independence and logistic regression. Only non-missing data was used and statistical significance was identified as a *p*-value equal to or <0.05. U.S. Census Bureau demarcation for region of the country (64) was used and programs outside the continental U.S. were coded as the South.

Our chi-square analysis examined if each of: (a) the prevalence of vegetarian and vegan nutrition instruction, (b) the belief that vegetarian and vegan nutrition should be taught, and (c) the connection between vegetarian and vegan diets and climate change and resource conservation varied significantly by (i) region of the country, (ii) type of ACEND-accredited program, (iii) ownership (public or private), or (iv) religious affiliation. Logistic regression was also used to examine if responses to the questions in (a), (b), and (c), above can be predicted from the variables ACEND-accredited program, region, ownership, and religious affiliation taken together.

Eighteen (18) questions offered the ability to write-in additional information as desired to prevent inadequate or missing information. Those responses were systematically categorized according to content. The results associated with these responses will be presented in a forthcoming publication specifically addressing curricular practices and demonstrated innovations in teaching vegetarian and vegan nutrition.

### Defining Vegetarian and Vegan Nutrition

While derivations of the term “plant-based diet” are commonly used nowadays within the food industry, healthcare, and the dietetics profession not to mention among consumers nationwide, a practice-based definition as well as one that is recognized by the Academy of Nutrition has not been realized. Thus, we use the standard practice-based terms “vegetarian diet” and “vegan diet” to specify not only the nutritive components of the respective dietary pattern but to recognize the possibility of nutrient deficiencies. A vegetarian diet was defined as a dietary pattern that is devoid of all flesh foods (meat, fowl, seafood, etc.) but may include eggs and dairy while a vegan diet was defined as dietary pattern devoid of all flesh foods as well as eggs, dairy, and other animal products (7).

## Results

The overall response rate was 36% with *n* = 205 program directors participating (*N* = 574 programs). DI programs (*n* = 97) encompassed the largest percentage (48%) of those responding to the survey; they also represent the largest number of ACEND-accredited programs. Table 1 shows the response rate among program types and the number of programs in the U.S. in 2017 whereas Table 2 shows the states that offered the greatest number of responses. Those five states comprise ~37% of programs in the U.S. Nearly 75% of respondents represented public institutions. Of those from private institutions (*n* = 51), 12% had a religious affiliation that are listed in Table 3.

**Table 1:** ACEND-accredited program response rate per type and number of programs in the U.S.

**Type of ACEND program**	**Number of programs in 2017**	**Number of responses**	**Response rate (%) per program type**	**Percentage of sample (%)**
Coordinated Program in Dietetics (CPD)	56	15	26.8	7.4
Dietetic Internship (DI)	249	97	38.9	47.6
Didactic Program in Dietetics (DPD)	223	82	36.7	40.2
Dietetic Technician Program (NDTR)	42	10	23.8	4.9

**Table 2:** States with 10 or more responses.

**State**	**Number of responses**	**Number of programs in that state**	**Percentage of respondents per number of programs in the state**
California	18	45	40.0
Illinois	12	26	46.2
New York	15	39	38.5
Ohio	13	35	37.1
Pennsylvania	10	26	38.5
Texas	18	44	40.9

**Table 3:** Responses from programs with a religious affiliation.

**Religion**	**Number of responses**
Baptist	1
Catholic	13
Church of Christ	1
Lutheran	2
Mormon	2
Nazarene	2
Presbyterian	1
Seventh-day Adventist	3
“Non-specific religious affiliation”	1

Over 51% (*n* = 105) of respondents state that vegetarian nutrition is taught. There was a significant difference (*p* = 0.00005) between the type of ACEND-accredited program and prevalence of teaching vegetarian nutrition, as indicated in Table 4. Vegetarian nutrition was taught in 80% of NDTR programs and in ~25% of DIs. When vegetarian nutrition is not taught, nearly 77% of respondents offer resources for students as needed for patient care and other circumstances.

**Table 4:** The prevalence of vegetarian nutrition instruction in ACEND programs.

**Prevalence**	**CPD**	**DPD**	**DI**	**NDTR**	**All**
Percentage responding “yes” to teaching vegetarian nutrition	73.3%	75.6%	24.7%	80.0%	51.5%
	(11/15)	(62/82)	(24/97)	(8/10)	(105/204)

*X^2^ = 53.0, p < 0.00005*.

Almost 49% of program directors state that vegan nutrition is taught. However, the percentage indicating that vegan nutrition was taught varied significantly between type of ACEND-accredited programs (*p* = 0.00005) as indicated in Table 5. For example, 90% of NDTR programs indicate that they teach vegan nutrition while only 23% of DI programs do. Beyond the statistically significant difference in prevalence of vegetarian and vegan nutrition instruction by program type, no other comparison (by region of country, program ownership, or religious affiliation) came remotely close to statistical significance.

**Table 5:** The prevalence of vegan nutrition instruction in ACEND programs.

**Prevalence**	**CPD**	**DPD**	**DI**	**NDTR**	**All**
Percentage responding “yes” to teaching vegan nutrition	73.3%	70.9%	22.6%	90.0%	49.2%
	(11/15)	(56/79)	(21/93)	(9/10)	(97/197)

*X^2^ = 51.4, p < 0.00005*.

Over 90% of program directors strongly agree (48%) and agree (43%) that vegetarian nutrition should be taught while nearly 44% strongly agree and 43% agree that vegan nutrition should be taught. Approximately 9% are unsure that either of these topics should be taught. Only one (*n* = 1) respondent disagreed that vegetarian and vegan nutrition should be taught. The percentage of respondents in the South (75.4%) who strongly agree or agree that vegan nutrition should be taught is notably smaller than the corresponding percentages for Northeast programs (96.0%), Midwest programs (88.6%), and Western programs (93.3%). These differences are statistically significant (*p* = 0.006).

Approximately 90% of program directors rate students' attitudes toward vegetarian and vegan diets as highly acceptable (36%) and acceptable (53%). Sixteen percent (16%) of respondents perceive barriers in addressing vegetarian and vegan nutrition in their respective programs with 67% citing time constraints *(n* = 21). Four (*n* = 4) respondents (~13%) cite knowledge deficits among staff.

Nearly 58% of respondents indicate that there is a vegetarian or vegan faculty member in their respective program; however, almost 69% who responded affirmatively do not believe their presence influences the inclusion of vegetarian and vegan nutrition in their respective curriculums.

Fifty percent (50%) of respondents (*n* = 93) identify the connections between vegetarian and vegan diets in climate change mitigation, resource conservation, and reducing impact on the natural environment while nearly 33% (*n* = 61) do not and nearly 17% are unsure.

## Discussion

To our knowledge, this is first study published of the prevalence of vegetarian and vegan nutrition instruction across ACEND-accredited programs in the U.S. and the first account of dietetics program educators linking vegetarian and vegan diets to climate change mitigation, resource conservation, and reducing impact on the natural environment. Several important findings follow from the study: (1) More than 90% of program directors that responded agree that vegetarian and vegan nutrition should be taught and perceive that students' attitudes toward vegetarian and vegan diets are favorable; (2) over half of programs (51%) teach vegetarian nutrition while slightly less (49%) teach vegan nutrition; (3) significant differences exist between the type of ACEND-accredited program and the prevalence of vegetarian (*p* = 0.00005) and vegan (*p* = 0.00005) nutrition instruction, respectively; (4) over 50% of programs connect vegetarian and vegan diets to climate change mitigation, resource conservation, and reducing impact on the natural environment; (5) region of the country (*p* = 0.006) impacted program directors' belief that vegan nutrition should be taught (*p* = 0.006).

It is clear that program directors support vegetarian and vegan nutrition instruction and perceive that students are also interested. This is reassuring given the burgeoning array of evidenced-based data that demonstrate the human health benefits of vegetarian and vegan dietary patterns, the benefits of diets high in fiber and nutrient-dense whole plant foods, and the concurrent positive impact on the natural environment. Overall, however, there appear to be discrepancies between enthusiasm for vegetarian and vegan diet instruction and incorporating these topics into dietetics education and training. Interestingly, most respondents did not perceive barriers in addressing vegetarian and vegan nutrition in their respective program.

While the literature is altogether insufficient with regard to data on incorporating vegetarian and vegan nutrition into dietetics education and training programs, the results of this study offer a compelling reason to formally include vegetarian and vegan nutrition training in accredited dietetics programs. Hence, the Vegetarian Nutrition (VN) Dietetic Practice Group (DPG) (65) of The Academy of Nutrition and Dietetics could be an invaluable asset in this realm, identifying the needs of ACEND-accredited program directors and offering resources that could increase self-efficacy among program directors and students while advancing the dietetics profession. Dietetic practice groups (DPG's) are professional interest groups of the Academy of Nutrition and Dietetics where a wealth of knowledge and skill about a distinct topic is shared among members (66). Additionally, the Academy of Nutrition and Dietetics' Vegetarian Nutrition Certification Program could prove invaluable (67).

The type of ACEND-accredited program predicts agreement that vegetarian nutrition instruction should be taught (*p* = 0.010). This may be due to differences in the perception of vegetarian nutrition as “foundational” knowledge as would be the case in DPD, CPD, and NDTR programs compared to a perception of the “applied” knowledge of DIs. The ACEND Accreditation Standards do not mandate vegetarian and vegan nutrition instruction as a requirement but do mandate competencies for health promotion and disease prevention that would encompass vegetarian and vegan diet instruction. For instance, there are mandates that the student would demonstrate competencies that “Develop and deliver products, programs or services that promote consumer health, wellness and lifestyle management” (68). Because of the preventive and therapeutic role of vegetarian and vegan diets in human health, mandating vegetarian and vegan diet training requirements by ACEND is advised. Furthermore, as plant-based foods are leading foodservice trends and meatless meals are becoming increasingly prevalent in food service establishments including healthcare and correctional facilities (69) and schools, mandatory vegetarian and vegan nutrition training for dietetics students that can be applied across population groups and ethnicities would better serve the needs of the general public and industry.

Because of the varying routes of education and training and the multitude of diverse populations served during the applied training experiences—there is no “one size fits all” approach to teaching and promoting vegetarian and vegan diets in practice. Our results suggesting that DIs have the lowest prevalence of vegetarian and vegan nutrition instruction deserves special attention. DIs comprise an important route of dietetics training and are based on competency requirements vs. the core knowledge *and* competency requirements of supervised practice programs (70). The DI is an invaluable applied training experience where dietetic interns could utilize vegetarian and vegan dietary principles in a multitude of ways such as direct patient care, rotations in public health and foodservice, cooking demonstrations, health promotion, or research. While students may come to their DI with textbook knowledge of vegetarian and vegan diets, formal instruction on applying vegetarian and vegan diets during the DI could improve patient outcomes and is therefore advisable.

Climate change mitigation goals will not be met without substantial adherence to plant-based consumption patterns (42), and 50% of respondents identify that their program connects the use of vegetarian and vegan diets in climate change mitigation, resource conservation, and reduced environmental impact. Given the ACEND curriculum mandate (starting July 1, 2017) that dietetics students “propose and use procedures as appropriate to the practice setting to promote sustainability, reduce waste and protect the environment,” (68) it is concerning that only 50% of program directors connect vegetarian and vegan diets to climate change mitigation, resource conservation, and reduced environmental impact.

Other studies identify similar trends. Webber and Sarjahani's (53) study of DI programs found that nearly 50% of directors either included content related to sustainable food systems or planned to (53). A 2011 survey of nutrition educators in dietetic training programs found that only 42% of respondents stated confidence in teaching the concepts of sustainable food systems (50). A lack of self-efficacy appears to be a barrier in teaching sustainable food systems in dietetics education (50) while inconsistent connections between diet and sustainability in training programs affects practice-based behaviors. For example, a 2017 survey of dietitians found that only 47% incorporated principles of sustainable food systems into practice (71).

Hence, an opportunity exists for the Hunger and Environmental Nutrition (HEN) DPG *and* the VN DPG to collaborate and offer guidance to ACEND program directors that would increase self-efficacy and eliminate knowledge deficits in this area. HEN's mission is to, “Empower members to be leaders in sustainable and accessible food and water systems (72). Although both DPGs have advocated for plant-based diets to reduce environmental impact (48, 73), an opportunity exists to strengthen collaboration, outreach, and impact.

Increased self-efficacy with regard to vegetarian and vegan nutrition and sustainable food systems impacts practice behaviors. Vegetarian dietitians and vegan dietitians were significantly more likely than non-vegetarian and non-vegan dietitians to use diet as a climate change mitigation strategy in practice, likely due to high levels of self-efficacy by virtue of the lived experience (30). Dietitians that engaged in the personal pro-environmental behaviors of (1) consuming organic foods (2) purchasing locally produced foods (3) consuming seasonal foods (4) growing produce and (5) composting food waste were significantly more likely to recommend the same behaviors in practice than those dietitians that do not engage in these behaviors (74, 75).

The percentage of program directors agreeing that vegan nutrition should be taught varied significantly by region of the country (*p* = 0.006); in particular, programs in the South agreed at notably smaller rates than the other three regions. Hawkins et al. found that dietitians residing in the South were significantly less likely to agree that climate change is an important issue than dietitians residing in other regions of the country (30). While these are separate issues, it points to regional differences among dietitians that are worthy of further exploration.

While over 200 program directors participated in this study, the results cannot be extrapolated to all program directors and settings. There are other limitations to consider. Those that completed this survey (36%) may be different and more supportive of vegetarian and vegan nutrition than those that did not respond (64%). It also could be that those that responded to the survey may have offered socially desirable responses.

In our survey, we used the terms vegetarian diets and vegan diets as they are defined in the evidenced-based literature and in dietetics practice (7). We could not locate research that clarifies how dietitians perceive the term “plant-based diet” which has not yet been defined in dietetics practice although it now used frequently the literature (76) and among the general public. Thus, it is unknown if we could we have garnered a higher response rate if framing our study with the term “plant-based diet” vs. vegetarian diet or vegan diet.

We did not inquire if ACEND-accredited programs were land-grant universities and colleges which could have influenced our response rate or the responses we received altogether. Land-grant institutions receive support to both teach and research agriculture and that may include animal husbandry and dairying (77).

The response to our survey was slightly lower than anticipated. However, our response rate of 36% was higher than other national surveys of dietitians (30, 60–62, 71). Our survey was initially sent in the summertime, which may have been a time where program directors were away from campus, on vacation, or contending with other program issues such as accreditation site visits. Although we were able to increase participation by way of reminder emails, we are not certain that all intended recipients received our email. Lastly, complimentary research methods such as focus groups could prove useful in future iterations of this research to expound upon the concepts of self-efficacy or the perceived knowledge deficits unveiled in this study.

## Conclusions

Most directors of ACEND-accredited programs are in favor of teaching students about vegetarian and vegan nutrition and believe students are receptive to such instructions. Despite this, formal instruction occurs in approximately half of programs overall while only half of program directors connect vegetarian and vegan diets to reduced environmental impact. These results suggest the need for novel interventions such as creating curriculum mandates for vegetarian and vegan nutrition instruction and increasing self-efficacy related to connecting diet to environmental impact. Importantly, increased collaboration among dietetics professionals could correct these deficits.

## Data Availability

The raw data supporting the conclusions of this manuscript will be made available by the authors, without undue reservation, to any qualified researcher.

## Ethics Statement

This study was carried out in accordance with the recommendations of The University of Massachusetts Amherst Institutional Review Board with written informed consent from all subjects. All subjects gave written informed consent in accordance with the Declaration of Helsinki. The protocol was approved by the the University of Massachusetts Amherst Institutional Review Board.

## Author Contributions

IH, AM, RG, and RW designed the survey. IH and AM collected the data. RG conducted the analysis with input from IH. IH and AM wrote the first draft with contributions from RG. All authors reviewed and commented on subsequent drafts of the manuscript.

### Conflict of Interest Statement

The authors declare that the research was conducted in the absence of any commercial or financial relationships that could be construed as a potential conflict of interest.

## Practice Implications

This is the first published study on the prevalence of vegetarian and vegan nutrition instruction in nationally-accredited dietetic programs in the U.S. Dietetic programs train future registered dietitian nutritionists and dietetic technicians. There is a strong connection between vegetarian and vegan diets and reduced risk of many nutrition-related chronic diseases. Additionally, these diets are associated with a reduced environmental impact. Despite this strong diet-disease and diet-environment connection, prior to this study, it was not known if dietetics students are taught the principles of vegetarian and vegan nutrition. This research provides information on both program directors' support for the inclusion of vegetarian and vegan nutrition instruction while demonstrating a gap between beliefs and practice behaviors. In an era of environmental concern, half connect use of a vegetarian or vegan diet to reduced environmental degradation. Using novel techniques and improved collaboration to increase the prevalence of vegetarian and vegan nutrition instruction and its application in dietetics training could better prepare Registered Dietitian Nutritionists and Dietetic Technicians to improve both individual health and planetary health outcomes.
